# Question Answering for Electronic Health Records: Scoping Review of Datasets and Models

**DOI:** 10.2196/53636

**Published:** 2024-10-30

**Authors:** Jayetri Bardhan, Kirk Roberts, Daisy Zhe Wang

**Affiliations:** 1 Department of Computer and Information Science and Engineering University of Florida Gainesville, FL United States; 2 School of Biomedical Informatics The University of Texas Health Science Center at Houston Houston, TX United States

**Keywords:** medical question answering, electronic health record, EHR, electronic medical records, EMR, relational database, knowledge graph

## Abstract

**Background:**

Question answering (QA) systems for patient-related data can assist both clinicians and patients. They can, for example, assist clinicians in decision-making and enable patients to have a better understanding of their medical history. Substantial amounts of patient data are stored in electronic health records (EHRs), making EHR QA an important research area. Because of the differences in data format and modality, this differs greatly from other medical QA tasks that use medical websites or scientific papers to retrieve answers, making it critical to research EHR QA.

**Objective:**

This study aims to provide a methodological review of existing works on QA for EHRs. The objectives of this study were to identify the existing EHR QA datasets and analyze them, study the state-of-the-art methodologies used in this task, compare the different evaluation metrics used by these state-of-the-art models, and finally elicit the various challenges and the ongoing issues in EHR QA.

**Methods:**

We searched for articles from January 1, 2005, to September 30, 2023, in 4 digital sources, including Google Scholar, ACL Anthology, ACM Digital Library, and PubMed, to collect relevant publications on EHR QA. Our systematic screening process followed PRISMA (Preferred Reporting Items for Systematic Reviews and Meta-Analyses) guidelines. A total of 4111 papers were identified for our study, and after screening based on our inclusion criteria, we obtained 47 papers for further study. The selected studies were then classified into 2 non–mutually exclusive categories depending on their scope: “EHR QA datasets” and “EHR QA models.”

**Results:**

A systematic screening process obtained 47 papers on EHR QA for final review. Out of the 47 papers, 53% (n=25) were about EHR QA datasets, and 79% (n=37) papers were about EHR QA models. It was observed that QA on EHRs is relatively new and unexplored. Most of the works are fairly recent. In addition, it was observed that emrQA is by far the most popular EHR QA dataset, both in terms of citations and usage in other papers. We have classified the EHR QA datasets based on their modality, and we have inferred that Medical Information Mart for Intensive Care (MIMIC-III) and the National Natural Language Processing Clinical Challenges datasets (ie, n2c2 datasets) are the most popular EHR databases and corpuses used in EHR QA. Furthermore, we identified the different models used in EHR QA along with the evaluation metrics used for these models.

**Conclusions:**

EHR QA research faces multiple challenges, such as the limited availability of clinical annotations, concept normalization in EHR QA, and challenges faced in generating realistic EHR QA datasets. There are still many gaps in research that motivate further work. This study will assist future researchers in focusing on areas of EHR QA that have possible future research directions.

## Introduction

### Motivation

Medical question answering (QA) may use biomedical journals, internet articles, and patient-specific data, such as that stored in the electronic health record (EHR), for QA. While there has been a great deal of work in medical QA [1-5], much of it does not help to answer patient-specific questions. In patient-specific QA, the answer is obtained from the patient’s medical record (ie, the EHR). This differs from other medical QA tasks due to linguistic issues (eg, EHR notes are very different in terminology, grammar, style, and structure from biomedical articles) and privacy limitations (eg, most biomedical articles have a publicly available abstract while there are laws in most countries limiting the sharing of patient records). In addition, patient-specific QA also prevents the use of many common QA techniques (such as aggregating answers from different biomedical articles to give weight to a consensus opinion). All this merits the review of EHR QA separate from other medical QA approaches to properly scope its data and methods. In this review paper, our aim is to discuss all the recent approaches and methodologies used for QA on EHRs. There have been some reviews on medical QA [6,7], but none of the previous review papers have focused solely on EHR QA. To the best of our knowledge, this is the first work that does a scoping review of QA on EHRs and examines the various datasets and methodologies used in EHR QA. There are several aspects of EHR QA that merit analysis of scope.

One such aspect is data modality and the variety of methodological approaches available for EHR QA. The methodological approach used is determined by the format of the EHR data. EHRs contain structured and unstructured data. Structured EHR data are based on standardized terminologies and ontologies and are often available in the form of relational databases. By contrast, unstructured EHR data have minimal standardization and include data types such as textual notes and clinical imaging studies. Two kinds of approaches are used for QA on structured EHR data. In the first approach [5], the natural language questions are converted into structured queries (such as SQL). These queries are used to retrieve answers from the database. In the second approach [8], the structured EHR tables are converted into knowledge graphs, following which the natural language questions are converted into graph queries (such as SPARQL) to extract answers from the database. QA on unstructured clinical EHR notes is mostly performed as a reading comprehension task, where given a question and clinical notes as context, a span of text from the notes is returned as the answer. There can also be multimodal EHR QA, which can use both structured and unstructured EHR data for QA. The aim of this study is to identify the studies that use EHR QA. We have further narrowed our search to EHR QA studies that use natural language processing (NLP) techniques on the questions but may or may not use NLP on the answers. We have excluded studies in which questions are asked about images (eg, radiology scans). as these questions and datasets have an entirely different focus. While QA over medical images is also a critical area of research, focusing a systematic review specifically on QA over EHR text (ie, structured and unstructured EHR containing textual information) allows a more detailed, manageable, and methodologically consistent study. This focused approach can yield deeper insights and more practical recommendations for improving QA systems on structured and unstructured data in health care settings.

The second aspect of EHR QA is the access to raw medical data. Due to privacy restrictions on clinical data, the replication and sharing of methods have been reduced compared with QA in other domains. This has led to the emphasis on sharable EHR datasets on which QA benchmarks can be made. Medical Information Mart for Intensive Care (MIMIC; MIMIC-IV) [9] and the eICU Database [10] are large publicly available EHR databases for patients admitted to intensive care units. The MIMIC-III [11] database provides the foundation for many of the existing QA studies on EHRs. MIMIC-IV introduced in the year 2020 is a recent update to the MIMIC-III database. Finally, the National NLP clinical challenges (n2c2) datasets (previously known as Informatics for Integrating Biology and the Bedside ie, i2b2 datasets) are another repository of clinical notes that have been used by the clinical QA community to develop EHR QA datasets.

Another aspect that warrants a scoping review of EHR QA is to study its different applications, including information extraction, cohort selection, and risk score calculation. For instance, Datta and Roberts [12] used a 2-turn QA approach to extract spatial relations from radiology reports. Similarly, Xiong et al [13] used a QA approach with the help of a machine-reading comprehension (MRC) framework for cohort selection, where every selection criterion is converted into questions using simple rules. For example, the selection criteria “ALCOHOL-ABUSE” is converted to the question “Current alcohol use over weekly recommended limits?” Following this, state-of-the-art MRC models such as Bidirectional Encoder Representations from Transformers (BERT) [14], BiDAF [15], BioBERT [16], NCBI-BERT, RoBERTa [17], and BIMPM [18] are used to match question and passage pairs to select cohorts. Furthermore, Liang et al [19] demonstrate that QA over EHR data can improve risk score calculation.

Finally, EHR QA systems face a variety of challenges ranging from parsing natural language questions to retrieving answers. For structured data, the natural language question needs to be parsed and converted to a structured query which can be used to query, the database. Medical terms from the queries, such as *blood pressure* and *leukemia*, must be normalized into standard ontologies. Clinical text frequently uses acronyms for medical concepts. These abbreviations are often ambiguous (eg, *pt* can refer to the patient or physical therapy) [20] and so must be identified and standardized by the QA system before querying over the EHR database or clinical data. These problems are exacerbated by the fact that the standard NLP approaches to such issues require large amounts of labeled data from the domain of interest. Few such labeled EHR datasets exist. This is because annotating EHR QA datasets requires clinical expertise and is time-consuming. Existing general-domain QA systems provide erroneous results when they are not trained on clinical QA datasets. In addition, most of the data found in EHRs are complex and contain both missing and inconsistent information [21,22], which adds to the difficulty of performing QA on EHRs. In the Discussion section, we have provided more detailed explanations of the various challenges of using QA on EHRs.

The wide variety of challenges and barriers discussed earlier motivates the need for a systematic scoping review of EHR QA literature. This paper identifies the articles that fall under the scope of EHR QA, identifies the difficult challenges faced in the task, and then enumerates both the data sources and QA methods that have been used to overcome such challenges. Finally, this paper also highlights the open issues in this field that demand future work in EHR QA.

### Template-Based Dataset Generation

**Figure 1:**
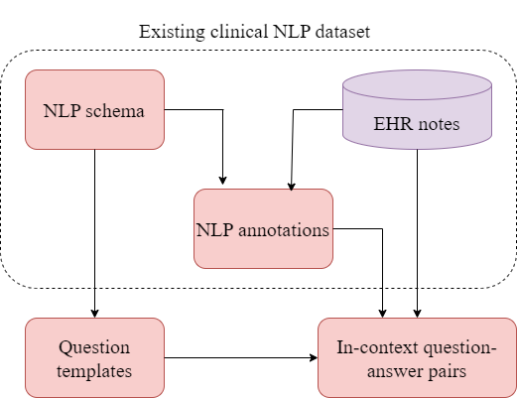
Flowchart showing the process of template-based dataset generation. The dotted boundary shows the existing non-question answering (QA) natural language processing (NLP) dataset along with the electronic health record (EHR) data. Question templates (and logical form templates) are constructed based on the schema of the EHR data. Clinical expert annotations of non-QA tasks based on the same EHR data are used to slot-fill placeholders in question templates and generate QA pairs.

Before diving into the methodology and results of this review, it is helpful to introduce a common semiautomated approach for building EHR QA datasets, as all large EHR QA datasets use this approach. This also impacts the screening process described in the Methods section. While other methods, such as semantic parsing with grammar-based techniques, exist for generating EHR QA datasets [23,24], template-based dataset generation remains the most widely used approach. In general, large EHR QA datasets are often required to increase the performance of EHR QA models. However, the creation of these datasets necessitates subject expertise. The slot-filling approach to generate template-based datasets is a semiautomated process, and hence very popular. Most of the EHR QA datasets are template-based [5,8,25-27]. The steps to construct template-based QA datasets are illustrated using a flowchart in Figure 1.

To minimize the need for clinical experts’ involvement in the dataset generation process, existing annotations of other non-QA clinical tasks (such as entity recognition and relations learning) are used for generating EHR question-answer pairs. The existing clinical annotations are used as proxy-expert in the dataset generation process [26]. In the first step, template questions containing placeholders (in place of entities) are constructed. An example of a question template is “Has this patient ever been treated with |medication|?” Here, |medication|, |problem|, and |treatment| are some commonly used placeholders. These placeholders in the questions are then slot-filled to obtain QA pairs using the entities in the EHR data and database schema (for a structured EHR database) with the help of the existing annotations from the clinical NLP datasets. So, in a question template, such as “Has this patient ever been treated with |medication|?” entities such as “insulin” and “Tylenol” from the EHR database and clinical notes (sharing the same entity type as |medication|) are slot-filled in the question template to obtain questions, such as “Has this patient ever been treated with insulin?” and “Has this patient ever been treated with Tylenol?” Following this approach, the RxWhyQA [27] and DrugEHRQA [25] datasets use the existing annotations from the 2018 n2c2 corpus, and the emrQA and emrKBQA datasets use annotations from 6 clinical tasks from the n2c2 repository [28-33].

Some EHR QA datasets, such as emrQA and emrKBQA, have used logical form templates in their template-based generation methods. Logical form templates are predefined structured representations of questions that provide a human-comprehensible symbolic representation, linking questions to answers. These are used to map EHR schema or ontology to represent relations in the questions. While generating these datasets, logical form templates are annotated by clinical experts for different question templates. For example, for the question template “what is the dosage of |medication|?” the annotated logical form template for emrQA is “MedicationEvent (|medication|) [dosage=x].” If more than 1 question template maps to the same logical form template, then they are considered paraphrases of each other. In the emrQA dataset, clinical expert annotations of non-QA tasks, such as entity recognition, relation learning, coreference, and medication challenge annotations (in the n2c2 repository), were used to slot-fill placeholders in question and logical form templates, which in turn were used to generate answers. This is shown in Figure 1. For example, the medication challenge in the n2c2 repository has annotations for medications and their corresponding dosage (eg, medication is nitroglycerin and the dosage is 40 mg). This was used to generate instances of the question “what is the dosage of |medication|?” along with instances of its corresponding logical form “MedicationEvent(|medication|) [dosage=x].” The dosage value, that is, 40 mg is the answer to the question. Similarly, the heart disease challenge dataset contains temporal information and was used to derive temporal-reasoning related question-answer pairs. The emrKBQA dataset used the same question templates and logical form templates of emrQA, which were then slot-filled using entities from the MIMIC-III knowledge base (KB) [11]. The answers of the emrKBQA dataset are present in the table cells of the MIMIC-III KB. The entity types used in the placeholders are test, problem, treatment, medication, and mode. So far, the slot-filling QA dataset generation process has proven to be the most common method of generating EHR QA datasets. This is because, while some manual annotation from domain experts is necessary, most of the process is automated.

## Methods

### Search Process

This study aims to review existing research on QA over EHRs. This includes papers on EHR QA datasets, QA models, and various approaches proposed over the years. We included papers related to QA in the clinical domain, specifically in EHRs. Papers in which EHRs are not used have been excluded. In this review, we define QA as the task of automatically providing precise, relevant answers to user queries from EHR data. This involves understanding and processing EHR data to extract and deliver specific information. We distinguish QA from broader interactive systems, such as conversational agents, chatbots, and general information retrieval systems, which may involve multiturn dialogue and do not focus solely on providing direct answers to questions. The scope of this review is specifically on structured and unstructured data within EHRs due to the unique challenges and methodologies involved in processing natural language and structured information. While medical images (eg, computed tomography, magnetic resonance imaging, and x-ray) and physical signals (eg, electrocardiograms and photoplethysmography) are critical components of EHRs, the techniques required to analyze these data types differ significantly from those used in structured and unstructured EHR data. Thus, studies focused on these modalities are excluded to maintain a clear and manageable focus on text-focused QA over structured and unstructured EHR data.

Each of the data sources has been queried to search for papers with the title having at least 1 of the following keywords: “clinical,” “medical,” “patient,” “EHR,” EMR,” “Electronic Health Record(s),” or “Electronic Medical Record(s).” This should be used in combination with one or more of the keywords: “question answering,” “questions,” “text to SQL,” “reading comprehension,” “machine comprehension,” “machine reading,” or “queries.” The search was limited to the period from January 1, 2005, to September 30, 2023, to review only recent works. We removed the duplicate studies after this.

### Screening Process

We used a 2-step screening process. The first step involved reading the abstracts and titles of all the papers, including only papers that were about EHR QA. We also removed many irrelevant papers that focused on “clinical questions” and “patient questions” but did not use NLP. We also removed non research papers (such as PhD dissertations and books).

In the final stage of screening, a full-text review was used to screen the papers further. Papers that were about query engines and tools and which did not use natural language questions were removed. We excluded papers in languages other than English. We also removed papers that just had an abstract and did not contain full text. There were some papers that were about information retrieval systems not specifically QA. These were also excluded. Furthermore, we have excluded studies in which questions are asked about images or electrocardiograms, as these studies have an entirely different focus. After the 2-stage screening process, we performed forward snowballing that cited the previously included papers on Google Scholar.

For this study, all the authors (JB, KR, and DZW) jointly made the rules for inclusion and exclusion criteria that were used during the paper collection and screening process. On the basis of the rules decided, JB collected the papers and worked on the overall screening process. Papers that were borderline for inclusion were independently screened by KR and then resolved after discussion. The final list made during the full-text review process was again independently screened and reviewed by JB and KR, with conflicts being resolved after discussion.

## Results

### Search and Screening Results

**Figure 2:**
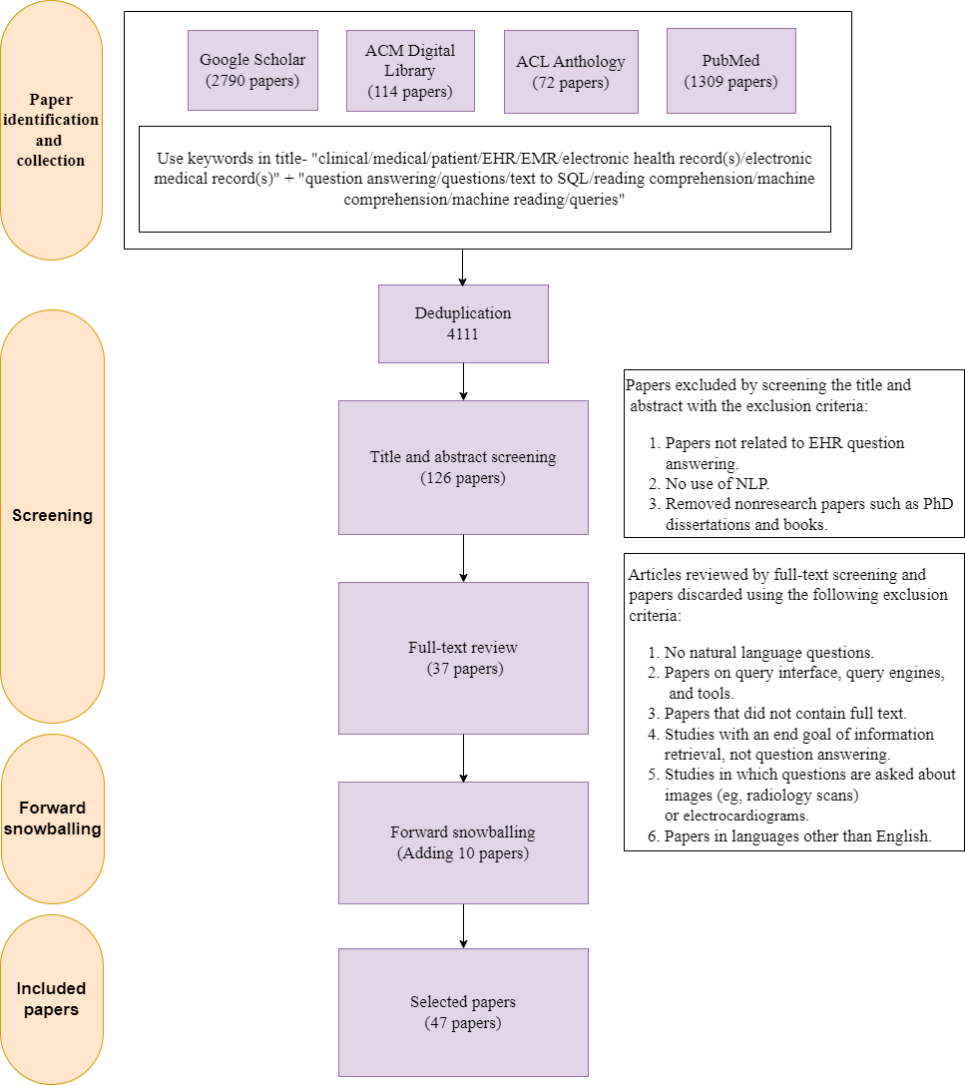
PRISMA (Preferred Reporting Items for Systematic Reviews and Meta-Analyses) diagram for study on question answering over electronic health records (EHRs). NLP: natural language processing.

We have fulfilled all PRISMA (Preferred Reporting Items for Systematic Reviews and Meta-Analyses) scoping review requirements and have attached a completed copy of the PRISMA checklist in Multimedia Appendix 1. The flowchart for conducting this study is shown in Figure 2.

In this record identification and collection step (ie, the search process), 4285 papers were collected (n=2790 from Google Scholar, n=114 from the ACM Digital Library, n=72 from the ACL Anthology, and n=1309 from PubMed). Following this, we removed the duplicate papers and obtained 4111 papers.

The first step of the screening process, the title and abstract screening step, yielded 126 papers. This was followed by the full-text review step, which yielded 37 papers. After the 2-stage screening process, we performed forward snowballing, adding 10 more papers to the list. We thus obtained 47 studies for EHR QA.

### Classification of Selected Papers

This section presents the findings of our study about existing EHR QA papers.

Table 1 lists our final list of selected publications post screening and then classified the papers based on their scope: “EHR QA datasets” and “EHR QA models.” We have further classified the studies on EHR QA models based on their function in the QA pipeline. “Full QA” denotes the papers on EHR QA models that are about end-to-end EHR QA systems. In the remaining part of the paper, we have provided our in-depth analysis of studies on QA using EHRs. In Multimedia Appendix 2, we have summarized our final list of selected papers.

**Table 1:** List of included papers in the systematic review and classification of selected papers based on their scope.

Type of study		References
EHR^a^ QA^b^ datasets		[5,8,23-27,34-51]
**EHR QA models**		
	Question generation	[43]
	Question paraphrasing	[52-54]
	Question classification	[55,56]
	Full QA	[5,8,25-27,38-42,48-51,57-73]

^a^EHR: electronic health record.

^b^QA: question answering.

**Figure 3:**
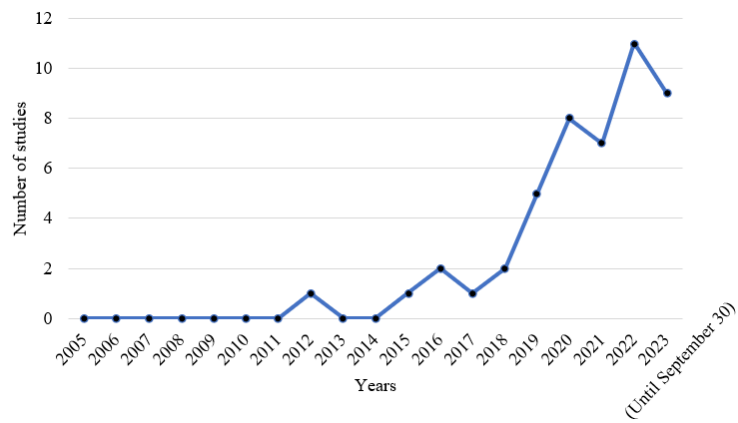
Number of studies on electronic health record question answering over the years. Since this systematic review is conducted based on studies published before September 30, 2023, hence the number of studies shown for the year 2023 is recorded only for a period of 9 months.

Figure 3 illustrates the number of publications on EHR QA over the years. From Figure 3, it can be observed that this is a relatively new field, and most of the publications in this domain are fairly recent. In the following subsections, we discuss our findings on existing EHR QA datasets, the various models used for questioning over EHRs, and also the different evaluation metrics used.

### Datasets

#### Dataset Classification and Analysis

Table 2 displays the total number of citations for all the EHR QA. It also lists the number of studies included in our review that have used these datasets. Moreover, Table 2 classifies the EHR QA based on the accessibility of the datasets. We can observe from the figures that emrQA [26] is the most popular out of all the other EHR QA datasets. This is likely due to emrQA’s size (1,295,814 question-logical forms and 455,837 question-answer pairs) and similarity to the Stanford QA dataset (SQuAD)-QA format.

**Table 2:** Popularity and accessibility of electronic health record (EHR) question answering (QA) datasets. We have listed the number of citations and the number of studies on EHR QA using the dataset. The information presented here is based on the data available on September 30, 2023.

Datasets	Number of citations	Number of studies on EHR QA using the datasets	Publicly available
emrQA [26]	151	11	Yes
MIMICSQL [5]	51	3	Yes
Yue et al [46]	40	0	No
MIMICSPARQL*^a^ [41]	27	2	Yes
Yue et al [42]	18	0	Yes
Roberts and Demner-Fushman [23]	s18	3	No
emrKBQA [8]	15	0	No
Raghavan et al [34]	13	0	No
Roberts and Demner-Fushman [24]	10	1	No
Soni et al [44]	7	3	No
Fan [35]	7	0	Yes
DrugEHRQA [25]	5	0	Yes
DiSCQ^b^ [43]	6	0	Yes
Oliveira et al [38]	3	0	No
RadQA^c^ [37]	3	1	Yes
EHRSQL [36]	3	0	Yes
Kim et al [39]	2	0	Yes
ClinicalKBQA^d^ [40]	2	0	No
Hamidi and Roberts [48]	1	0	No
MedAlign [49]	1	0	No
RxWhyQA [27]	0	0	Yes
Mishra et al [45]	0	0	No
CLIFT^e^ [47]	0	0	No
Mahbub et al [50]	0	0	No
Dada et al [51]	0	0	No

^a^This dataset follows the original schema of Medical Information Mart for Intensive Care (MIMIC-III).

^b^DiSCQ: Discharge Summary Clinical Questions.

^c^RadQA: Radiology Question Answering Dataset.

^d^ClinicalKBQA: Clinical Knowledge Base Question Answering.

^e^CLIFT: Clinical Shift.

**Figure 4:**
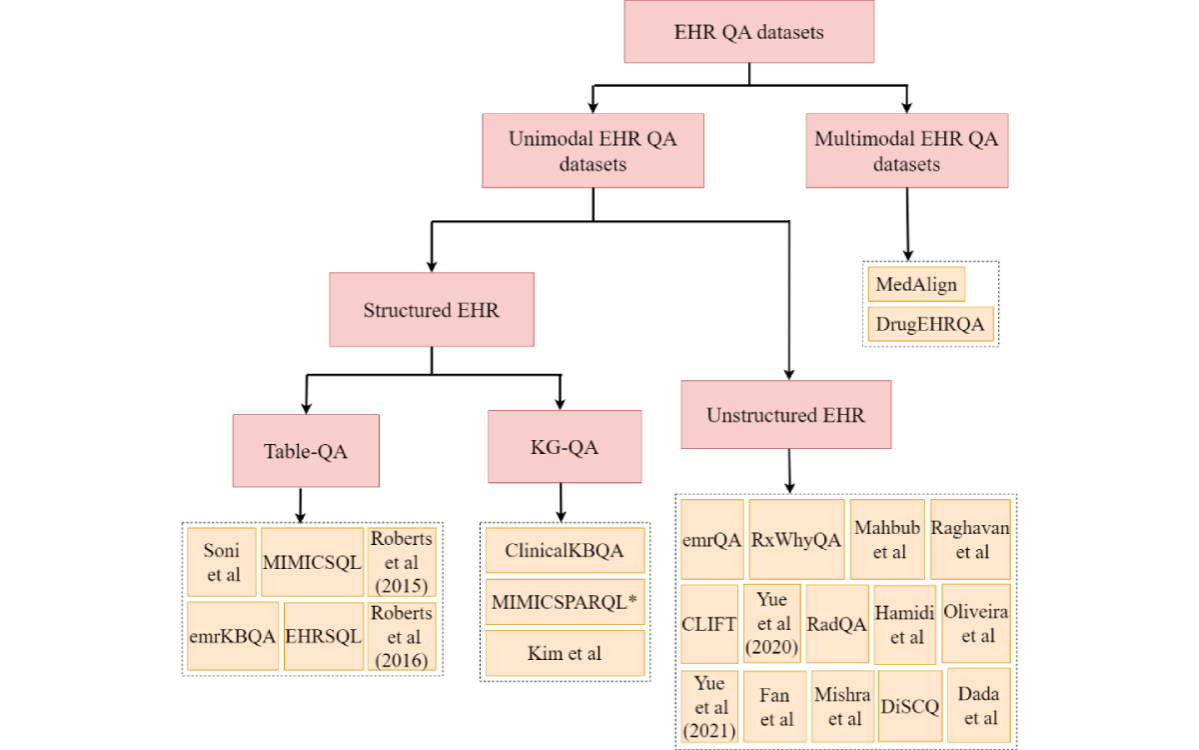
Classification of electronic health record (EHR) question answering (QA) datasets based on modality [5,8,23-27,34-51,74]. The datasets can be unimodal (based on structured or unstructured EHR data) or multimodal.

**Figure 5:**
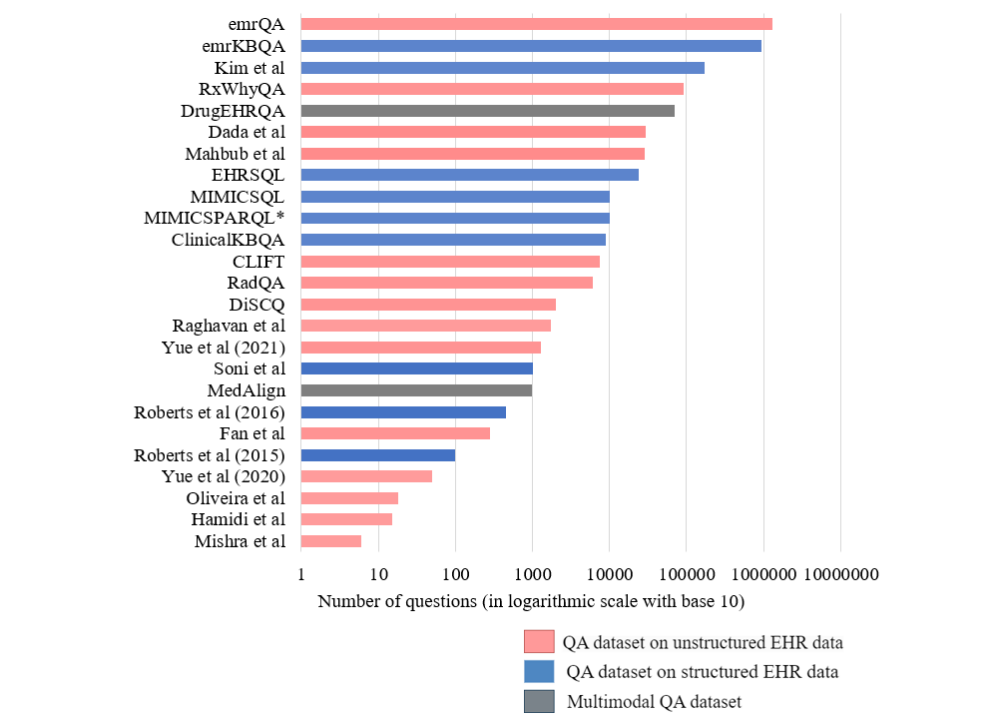
Plot of the total number of questions included in various electronic health record (EHR) question answering (QA) datasets and classification into unstructured, structured, and multimodal EHR QA datasets [5,8,23-27,34-51,74].

The classification of EHR QA datasets is shown in Figure 4. EHR QA datasets can be unimodal or multimodal. Unimodal EHR QA datasets are based on QA over 1 modality, which can be in the form of structured EHR data or unstructured EHR clinical notes. Multimodal EHR QA datasets use both modalities for QA over EHRs. The DrugEHRQA [25] and MedAlign [49] datasets are examples of multimodal EHR QA datasets that use structured and unstructured EHR data for QA. Figure 5 shows the size and modalities of the different EHR QA datasets.

It is to be noted that the dataset introduced in Mishra et al [45] uses 6 key questions (as can be observed from Figure 5), that is, the same 6 questions have been reused for all the articles. Multimedia Appendix 3 [5,8,23-27,34-51,74] summarizes the existing EHR QA datasets. The EHR databases or corpora contain answers to the questions. From the table in Multimedia Appendix 3, we can infer that most of the EHR QA datasets on structured EHR data use the MIMIC-III database [5,8,36,39,41], while most of the QA datasets on unstructured data use the n2c2 repository [26,27,35] or the clinical notes of MIMIC-III [37,42,43,45-48].

The following sections describe the QA datasets based on unimodal (structured or unstructured) and multimodal EHR data in detail.

#### QA Datasets Based on Unstructured EHR Data

Unstructured free text EHR data comprises discharge summaries, radiology reports, laboratory reports, medical images, progress notes, and many more note types. It accounts for roughly 80% of all EHR data [75]. One way to make use of this is to create a QA system that can extract answers from unstructured EHR data. Most of the QA datasets on unstructured clinical data are designed for the task of machine comprehension. Given clinical notes (containing patient information) and natural language questions, the objective of these tasks is to retrieve a span of text from the clinical notes as the answer.

The emrQA [26] is the most popular among the EHR QA datasets and contains 455,837 question-answer samples along with 1,295,814 question-logical form pairs. It relies on expertly annotated n2c2 datasets [28-33]. A semiautomatic, template-based process was used to generate the dataset. From Figure 5, we can observe that the emrQA is the largest EHR QA dataset overall.

Despite emrQA’s popularity, it has some flaws. The emrQA dataset has attempted to simulate clinicians’ questions using predefined templates and generating QA datasets by slot-filling with entities. Consequently, the questions in the emrQA dataset are not very realistic or relevant to the medical community. They are also highly repetitive. For example, it was shown in Yue et al [46] that the same model performance was obtained by sampling 5% to 20% of the dataset as with the entire dataset. This makes it necessary to create datasets that are more realistic and closer to real physicians’ questions. Later, Yue et al [42] developed 975 human-verified questions along with 312 human-generated questions based on 36 discharge summaries from MIMIC-III’s clinical notes. After randomly sampling 100 questions individually, the 975 human-verified questions and 312 human-generated questions, it was learned that 96% of the human-verified questions were obtained from the emrQA’s templates, and 54% of the human-generated questions of Yue et al [42] used the same templates from emrQA.

The RxWhyQA dataset [27] and Fan [35] dataset have reasoning-based questions. The RxWhyQA dataset contains a combination of reasoning-based unanswerable and multi-answer questions. Similar to the emrQA dataset, RxWhyQA is also a template-based dataset and hence not very realistic. This made it necessary to create datasets that are more realistic and closer to real physicians’ questions. The Discharge Summary Clinical Questions dataset [43] was created to address this issue and included questions about clinically relevant problems by gathering questions that clinicians could ask. It includes 2029 questions and >1000 triggers based on MIMIC-III discharge reports.

Most of the QA on unstructured EHR datasets is based on discharge summaries [26,27,35,43,45,74]. RadQA [37] and Dada et al [51] are the only 2 QA datasets that use radiology reports for QA. The types of questions used in the EHR QA datasets vary greatly from one another. emrQA covers different types of questions, including factual (“what” and “show me”), reasoning (“how” and “why”), and class prediction (“is” and “has”). However, the distribution of questions for the emrQA dataset is skewed; that is, most of the questions in the emrQA dataset start with “what.” In comparison, the authors of RadQA claim that the questions in their dataset are more evenly distributed than emrQA. The RxWhyQA dataset [27] and Fan [35] are reasoning-based questions, and hence their questions have “why cues.” Raghavan et al [34] predominantly have temporal questions along with questions on presence or absence (ie, “yes” or “no” questions) as well as questions on medications, tests, and procedures. Mishra et al [45], by contrast, restrict themselves to diagnosis-related questions. Table 3 compares some of the EHR QA datasets using unstructured EHR data for QA. Out of the 14 QA datasets on unstructured EHR notes, only 4 of them (RadQA [37], RxWhyQA [27], Hamidi and Roberts [48], and Dada et al [51]) contain unanswered questions.

**Table 3:** Comparison of different electronic health record (EHR) question answering (QA) datasets on unstructured data.

Dataset	Mode of dataset generation	Total questions, n	Unanswered questions, n	Average question length (tokens, n)	Total articles, n	Average article length (tokens, n)
emrQA [26]	Semiautomatically generated	1,295,814	0	8.6	2425	3825
RxWhyQA [27]	Automatically derived from the n2c2^b^ 2018 ADEs^c^ NLP^d^ challenge	96,939	46,278	—^a^	505	—
Raghavan et al [34]	Human-generated (medical students)	1747	0	—	71	—
Fan [35]	Human-generated (author)	245	0	—	138	—
RadQA^e^ [37]	Human-generated (physicians)	6148	1754	8.56	1009	274.49
Oliveira et al [38]	Human-generated (author)	18	0	—	9	—
Yue et al [42,74]	Trained question generation model paired with a human-in-the-loop	1287	0	8.7	36	2644
DiSCQ^f^ [43]	Human-generated (medical experts)	2029	0	4.4	114	1481
Mishra et al [45]	Semiautomatically generated	6 questions or article	—	—	568	—
Yue et al [46]	Human-generated (medical experts)	50	0	—	—	—
CLIFT^g^ [47]	Validated by human experts	7500	0	6.42, 8.31, 7.61, 7.19, and 8.40 for smoke, heart, medication, obesity, and cancer datasets	—	217.33, 234.18, 215.49, 212.88, and 210.16 for smoke, heart, medication, obesity, and cancer datasets, respectively
Hamidi and Roberts [48]	Human-generated	15	5	—	—	—
Mahbub et al [50]	Combination of manual exploration and rule-based NLP methods	28,855	—	6.22	2336	1003.98
Dada et al [51]	Human-generated (medical student assistants)	29,273	Unanswered questions available	—	1223	—

^a^Not applicable.

^b^n2c2: natural language processing clinical challenges.

^c^ADE: adverse drug events.

^d^NLP: natural language processing.

^e^RadQA: Radiology Question Answering Dataset.

^f^DiSCQ: Discharge Summary Clinical Questions.

^g^CLIFT: Clinical Shift.

#### QA Datasets Based on Structured EHR Data

EHR tables contain patient information, such as diagnoses, medications prescribed, treatments, procedures recommended, laboratory results details, and so on. It also includes a lot of temporal information, such as the date of admission, the date of discharge, and the duration of certain medications. The goal of QA tasks over structured databases is to translate the user’s natural language question into a form that can be used to query the database.

The QA task on structured EHRs can be classified into 2 types based on the 2 most common forms of structured data: relational databases and knowledge graphs. The first type of QA task entails converting natural language questions into SQL (or logical form) queries that can be used to query the database. In the other type of approach, the EHR data exist in the form of knowledge graphs containing patient information, and the natural language questions are often converted into SPARQL queries to retrieve the answer. MIMICSQL, emrKBQA, and EHRSQL are examples of datasets that use table-based QA approaches whereas datasets such as Clinical Knowledge Base Question Answering (ClinicalKBQA) and MIMIC-SPARQL* use knowledge graph–based QA approaches.

MIMICSQL [5] is a large dataset used for question-to-SQL query generation tasks in the clinical domain. The MIMICSQL dataset is based on the tables of the MIMIC-III database. emrKBQA [8] is the counterpart of the emrQA dataset for QA on structured EHRs. It is the largest QA dataset on structured EHR data (Figure 5) and contains 940,000 samples of questions, logical forms, and answers. EHRSQL [36] is a text-to-SQL dataset for 2 publicly available EHR databases—MIMIC-III [11] and eICU [10]. It is the only QA dataset on structured EHR data that contains unanswerable questions. Other QA datasets for structured EHR databases include MIMIC-SPARQL* [41] and ClinicalKBQA [40]. However, unlike previous table-based QA datasets, these are knowledge graph–based QA datasets.

**Figure 6:**
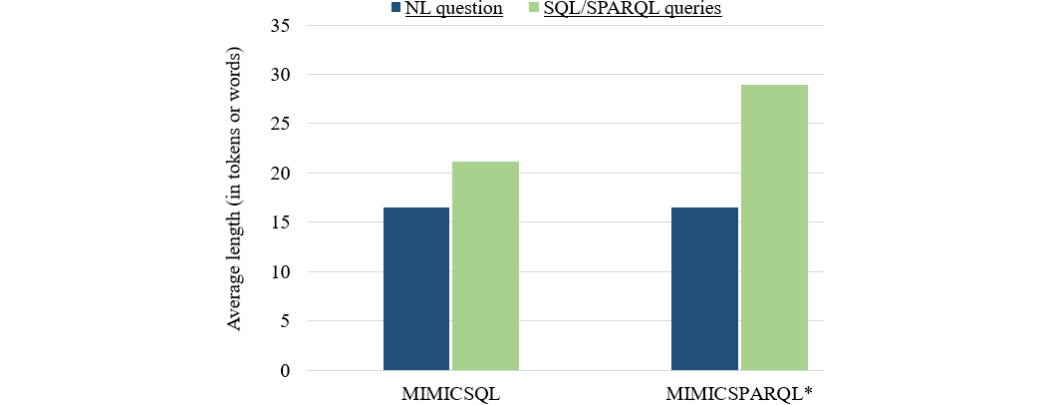
Average length of questions and SQL/SPARQL Protocol and RDF Query Language (SPARQL) queries (in tokens or words) for MIMICSQL and MIMICSPARQL datasets.

The MIMICSQL dataset [5] was created by making changes to the MIMIC-III database’s original schema. In total, 9 tables from the MIMIC-III database were merged into 5 tables to simplify the data structure. The derived tables and schemas were not the same as those found in actual hospitals and nursing homes. Therefore, a model trained on the MIMICSQL dataset will not be able to generalize to a real-world hospital setting. To address this issue, Park et al [41] introduced 2 new datasets—a graph-based EHR QA dataset (MIMIC-SPARQL*) and a table-based EHRQA dataset (MIMICSQL*). This was done to improve the analysis of EHR QA systems and to investigate the performance of each of these datasets. MIMICSQL [5] was modified to create MIMICSQL* to comply with the original MIMIC-III database schema [11]. The graph counterpart of the MIMICSQL* dataset is MIMIC-SPARQL*. Figure 6 compares the 2 datasets—MIMICSQL and MIMICSPARQL* based on the length of the questions and the length of SQL/SPARQL queries.

Wang et al [40] generated a clinical knowledge graph (ClinicalKB) with the help of clinical notes of n2c2 annotations and linked different patient information to perform KB QA. Furthermore, Wang et al [40] generated the ClinicalKBQA dataset that can answer statistics-related questions about different patients as well as questions specific to individual patient records.

Roberts and Demner-Fushman [23,24] and Soni et al [44] introduced datasets where logical forms (based on lambda calculus expressions) were created for questions to perform QA on EHR data (known as semantic parsing). Roberts and Demner-Fushman [23,24] generated a bottom-up grammar-based method that generates logical forms for question phrases. Soni et al [44] constructed the question-logical form dataset with the help of the Fast Healthcare Interoperability Resources server.

#### QA Datasets Based on Multimodal EHR Data

Multimodal QA is QA over >1 modality. QA over >1 modality can help in seeking more accurate answers while taking advantage of >1 source for QA. DrugEHRQA [25] is the first multimodal EHR QA dataset. It uses both structured tables of MIMIC-III and unstructured clinical notes for QA. The DrugEHRQA dataset is a template-based dataset containing medicine-related queries, its corresponding SQL queries for querying over multi-relational EHR tables, the retrieved answer from one or both modalities, as well as the final multimodal answer. The MedAlign dataset [49] also uses structured and unstructured EHR data for QA, but indirectly. The instructions and response pairs of the MedAlign dataset are based on XML markup documents that are derived from structured and unstructured EHR data.

### Models and Approaches for QA on EHRs

This section describes the various QA models used in EHRs. QA tasks vary depending on the EHR modality because different information is found in different modalities. Most QA models on clinical notes use a MRC approach, that is, for a given question, the QA model is trained to predict the span of text containing the answer from the clinical note [26,27,38,42,48,50,51,59,65,66]. For QA over EHR tables, translating questions to SQL queries is one of the major approaches used to retrieve answers from the EHR tables [5,61,62,71]. The other approach is to transform the EHR relational database into a knowledge graph and perform a knowledge-graph QA task [39,41,61]. Table 4 summarizes all the QA models (full QA) used for EHRs. Multimedia Appendix 4 [5,8,23,25-27,38-42,44,48-51,57-74] contains further information about these models.

**Table 4:** Summary of models for question answering (QA) over electronic health records.

Papers	Model
Pampari et al [26]	For QA task: DrQA’s document reader and a multiclass logistic regression model for predicting class. For question-to-logical form task: a sequence-to-sequence model is used with attention paradigm
Moon et al [27]	Clinical BERTa model with incremental masking
Oliveira et al [38]	BioBERTpt
Yue et al [42]	For QA task: DrQA’s DocReader and ClinicalBERT For question generation task: QPPb module is used with base question generation models (NQGc, NQG++, and BERT-SQGd)
Hamidi and Roberts [48]	ChatGPT (versions 3.5 and 4), Google Bard, and Claude
Fleming et al [49]	6 language models: GPT-4 (32 K tokens+multistep refinement), GPT-4 (32-K tokens), GPT-4 (2K tokens), Vicuña-13B (2K tokens), Vicuña-7B (2K tokens), and Vicuña-7B (2K tokens)
Mahbub et al [50]	Baseline models: 4 state-of-the-art pretrained language models—BERT, BioBERT, BlueBERT, and ClinicalBERT for QA. Modeling with transfer learning: sequential learning and adversarial learning
Dada et al [51]	G-BERT and GM-BERT
Roberts and Patra [57]	Hybrid semantic parsing method, uses rule-based methods along with a machine learning–based classifier.
Rawat and Li [59]	Uses multilevel attention layers along with local and global context while answering questions
Rawat et al [60]	Multitask learning with BERT and ERNIE [76] as the base model
Wen et al [64]	BERT model trained on different data sources
Soni and Roberts [65]	BERT, BioBERT, clinical BERT, and XLNet
Mairittha et al [66]	BERT (large, uncased, whole word masking), BERT fine-tuned on SQuADe benchmark, BioBERT, and an extended BioBERT fine-tuned on unstructured EHR data
Moon et al [67]	ClinicalBERT model fine-tuned on SQuAD-why dataset
Li et al [68]	Clinical-Longformer and Clinical-BigBird language model
Yang et al [69]	GatorTron language model
Lehman et al [73]	12 different language models (T5-Base, Clinical-T5-Base-Ckpt, Clinical-T5-Base, RoBERTa-Large, BioClinRoBERTa, GatorTron, T5-Large, Clinical-T5-Large, PubMedGPT, T5-XL, Flan-T5-XXL, and GPT-3)
Kang et al [70]	KALAf
Wang et al [5]	TREQSg
Raghavan et al [8]	Min et al [77] for sequence-to-sequence task along with ParaGen and ParaDetect model
Pan et al [62]	Medical text-to-SQL model
Soni and Roberts [63]	Tranx, Coarse2Fine, transformer, and lexicon-based
Tarbell et al [71]	T5 language model for question-to-SQL task, along with data augmentation method for back-translation
quEHRy [72]	End-to-end EHR QA pipeline with concept normalization (MetaMap), time frame classification, semantic parsing, visualization with question understanding, and query module for FHIRh mapping and processing
Kim et al [39]	Program-based model
Wang et al [40]	Attention-based aspect reasoning
Park et al [41]	Seq2Seq model [78] and TREQS [5]
Schwertner et al [58]	ENSEPROi framework
Bae et al [61]	Unified encoder-decoder architecture that uses input masking
Bardhan et al [25]	MultimodalEHRQA

^a^BERT: Bidirectional Encoder Representations from Transformers.

^b^QPP: question phrase prediction.

^c^NQG: Neural Question Generation.

^d^BERT-SQG: BERT-Sequential Question Generation.

^e^SQuAD: Stanford QA dataset.

^f^KALA: Knowledge-Augmented Language model Adaptation.

^g^TREQS: Translate-Edit Model for Question-to-SQL.

^h^FHIR: Fast Healthcare Interoperability Resources.

^i^ENSEPRO: Ensino de Serviços Proativos (in Portuguese), which translates to Teaching Proactive Services.

We can observe from Table 4 that over the years, DrQA’s document reader, BERT, and ClinicalBERT are some of the most popular QA models used for unstructured clinical notes [26,27,42,50,60,64-67]. However, since the year 2022, there has been a sharp rise in the number of studies introducing new large language models (besides BERT and other variants of BERT) for MRC tasks [48,68,69,73]. For example, Clinical-Longformer and Clinical-BigBird [68] and GatorTron [69] language models were proposed for various tasks, including EHR QA. Hamidi and Roberts [48] also evaluated the performance of ChatGPT, Google Bard, and Claude for EHR QA. Lehman et al [73] is another study introduced in the year 2023 that evaluated different language models (T5-Base, Clinical-T5-Base-Ckpt, Clinical-T5-Base, RoBERTa-Large, BioClinRoBERTa, GatorTron, T5-Large, Clinical-T5-Large, PubMedGPT, T5-XL, Flan-T5-XXL, and GPT-3) for MRC task on EHR notes.

For QA over structured EHR tables, Translate-Edit Model for Question-to-SQL (TREQS) [5], Medical text-to-SQL (MedTS) [62], and T5 [71] models are used. TREQS [5] is a sequence-to-sequence model that uses a question encoder to convert the questions into vector representations, which are then decoded into SQL queries by the decoder. The generated SQL queries are further edited using an attentive-copying mechanism and recovery mechanism. The MedTS model [62] is another text-to-SQL model that uses a pretrained BERT model as an encoder and a grammar-based long short-term memory (LSTM) decoder to obtain an intermediate sequence. Experiments on the MIMICSQL dataset have shown that the MedTS model outperforms the TREQS model by 22.8% logical form accuracy and by 24.5% execution accuracy. Note that logical form accuracy and execution accuracy are some common evaluation metrics in text-to-SQL tasks. They are explained in detail in the Evaluation Metrics section. Some other examples of table-based QA methods include Tranx [79], Coarse2Fine [80], transformer-based model [63], lexicon-based models [63], quEHRy [72], and sequence-to-sequence tasks used with ParaGen and ParaDetect models [8].

Some models for QA over graph-based EHR are the sequence-to-sequence model [41], TREQS model [41], UniQA model [61], and attention-based aspect reasoning method for KBQA [40]. For most of these models [41,61], the EHR relational database (such as MIMIC-III) is converted into a knowledge graph, and a question-to-SPARQL task is performed to retrieve answers from the knowledge graph. The sequence-to-sequence model [78] uses a bidirectional LSTM as the encoder and uses LSTM decoder while having an attention paradigm. Unlike the TREQS model [5], the sequence-to-sequence model cannot handle out-of-vocabulary words. The UniQA model [61] uses a unified encoder-decoder architecture along with input-masking and value-recovering techniques; thus, it is robust to typos and mistakes in questions. The condition value of the query generated using the question-to-query model is compared with the values in the database. This is called the condition value recovery technique. ROUGE-L score [81] is used to check the similarity between the values in the database to that of the condition values in the generated query. Then, the condition values are replaced with values most similar to those in the database. After applying the recovery technique, UniQA outperforms both the sequence-to-sequence model (by 74.6% logical form accuracy and 69.2% execution accuracy) and the TREQS model (by 14.2% logical form accuracy and 11.2% execution accuracy).

Most of the existing works discuss only QA on unimodal EHR data. Bardhan et al [25] have proposed a simple pipeline for multimodal QA on EHRs (called MultimodalEHRQA) that uses a modality selection network to choose the modality between structured and unstructured EHR as the preferred modality. If the selected modality obtained is “unstructured text,” then QA is performed over the clinical notes using BERT or ClinicalBERT, and the span of text from the clinical notes is returned as the multimodal answer. Similarly, if the preferred modality selected is “structured tables,” then a text-to-SQL task is performed using the TREQS model [5]. Further research is still needed to develop a multimodal QA model capable of handling the more challenging task of using answers from both structured and unstructured data to obtain a contextualized answer.

### Evaluation Metrics

In this section, we discuss the different evaluation metrics used for EHR. Evaluation metrics are used to evaluate the efficacy of different models. Multimedia Appendix 5 lists the different evaluation metrics used in different EHR QA studies.

The type of QA task would determine the evaluation metrics used. For QA with MRC tasks (eg, in QA over clinical notes), exact match and *F*_1_-score are the most popular metrics for evaluation [26,27,42,46,50,51,60,65,66,68]. Exact match refers to the percentage of predictions that exactly match the ground truth answers. In [26], an exact match is used to determine if the answer entity is included in the evidence. If not, it is determined whether the projected span of evidence is within a few characters of the actual evidence. Precision measures the number of tokens in a prediction that overlap with the correct answer compared with the total number of tokens in the prediction. Recall calculates the proportion of tokens in the correct answer that are included in the prediction compared with the total number of tokens in the correct answer. Precision and recall are represented using equations 1 and 2.

Precision=TP/(TP+FP) **(1)**

Recall=TP/(TP+FN) **(2)**

Where TP, FP, and FN represent true positives, false positives, and false negatives, respectively, at the token level. The *F*_1_-score is a broader metric that calculates the average overlap between the prediction and the correct answer [6]. It is defined as the harmonic mean of precision and recall. This is represented using equation 3. Wen et al [64] and Moon et al [67] used exact match and partial match to assess the QA models for answering questions based on patient-specific clinical text. The *F*_1_-score was used for weighing the partial match between the predicted token of words and the golden token of words. The *F*_1_-score is calculated using the following equation:

F1=2×(Precision×Recall) / (Precision+Recall) **(3)**

Evaluation metrics, such as logical form accuracy and execution accuracy, are commonly used for evaluating models responsible for table-based QA that use a question-to-SQL query-based approach [5,62,71]. They are also used for graph-based QA that uses a question-to-SPARQL query-based approach [41,61]. The logical form accuracy is calculated by making a string comparison between the predicted SQL/SPARQL queries and the ground truth queries, and execution accuracy is calculated by obtaining the ratio of the number of generated queries that produce correct answers to the total number of queries [5]. There are instances where execution accuracy might include questions where the generated SQL query is different from the ground truth query, but the returned answer is the same. Structural accuracy is another metric to evaluate models used for question-to-SQL/question-to-SPARQL query tasks [41,61]. Structural accuracy is similar to measuring logical form accuracy, except that it ignores the condition value tokens. Condition value refers to the string value or numeric value in the WHERE part of the SQL/SPARQL query. For example, in the SQL query “SELECT MAX(age) from patients WHERE Gender=‘F’ and DoB>2020,” “F” and 2020 are the condition values. The objective of using structural accuracy is to evaluate the accuracy of converting questions to SQL/SPARQL query structures, by not giving importance to the condition values (similar to the Spider dataset [82]). Raghavan et al [8] use exact match and denotation accuracy for evaluating clinical table-QA models. The framework involves the following 2 stages: (1) predicting logical forms for questions and (2) obtaining answers from the database with logical forms as input. Exact match is used for semantic parsing, while denotation accuracy is used to evaluate models for obtaining answers from logical forms. Denotation accuracy checks if the logical forms that are input to the model return the correct label answer, and the exact match is used to check if the logical forms generated are the same as the ground truth logical forms.

A variety of text-generating metrics have been used to evaluate question paraphrasing. Soni et al [52] used Bilingual Evaluation Understudy (BLEU) [83], Metric for Evaluation of Translation with Explicit ORdering (METEOR) [84], and translation error rate (TER) [85] for evaluating paraphrasing models. The BLEU score evaluates how closely generated paraphrases (or candidate translations) resemble those in the reference. This is done with exact token matching. The BLEU score is calculated as follows:

BLEU = brevity penalty×exp (∑ w_n_ log p_n_) **(4)**

where,

Bravity penalty=min(1, exp(1−reference length/output length )) **(5)**

p_n_=total number of candidate n-grams/total number of matched n-grams **(6)**

In equation 4, w_n_ represents the weight for each n-gram. The METEOR score, by contrast, uses synonyms and word stems. This is represented using the following equations:

METEOR = F×(1—Penalty) **(7)**

where,

F = (Precision × Recall)/(α × Precision + (1 − α) × Recall) **(8)**

Penalty = γ × (ch/m)β **(9)**

In equation 9, “ch” is the number of chunks that match, and m is the number of uniforms that match between the prediction and the reference. The parameters α, β, and γ are adjusted to maximize the correlation with human judgments. The edit distance (the number of edits necessary to change one sentence into another) between generated and reference paraphrases is measured by the TER score. It is calculated by adding up all the edits, dividing that total by the number of words, and multiplying that result by 100, that is,

TER = (number of edits / average number of reference words) × 100 **(10)**

## Discussion

### Challenges and Existing Solutions

#### Limited Number of Clinical Annotations for Constructing EHR QA Datasets

There are very few clinical EHR annotations that are publicly available. The n2c2 repository is one of the very few public repositories that hosts EHR NLP datasets (that can be used to create template-based QA datasets). This is because creating these annotations requires a lot of manual work, which can be time-consuming, and at the same time requires domain knowledge [25,26]. For the same reasons, it was difficult to annotate EHR QA datasets. There are also some ethical issues and privacy concerns that need to be handled while constructing EHR QA datasets. This involves the deidentification of information related to patients.

Datasets such as emrQA [26] and ClinicalKBQA [40] are examples of template-based datasets that have used the available expert annotations of the n2c2 repository to generate large-scale patient-specific QA datasets using semiautomated methods, taking advantage of the limited clinical annotations. While questions in these datasets do not represent the true distribution of questions one would ask to EHR, their scale makes them valuable for transfer learning and method development.

#### Concept Normalization in Clinical QA

QA in any domain has its own challenges. However, clinical QA has added challenges. One major challenge is when different phrases are used for the same medical concept in the question and the database. Clinical normalization is used to deal with this issue. Clinical normalization involves recognizing the medical entities and terminologies and converting them into a singular clinical terminology or language. Many EHR QA datasets, such as emrQA, have used MetaMap [86] during the dataset generation process to map medical terminologies mentioned in the clinical text to the Unified Medical Language System (UMLS) Metathesaurus. However, it has been argued that concept normalization for EHR QA is fundamentally different than the task on clinical notes [72], so QA-specific datasets are clearly needed.

#### Generating Realistic EHR QA Datasets

It is necessary to ensure that questions in EHR QA datasets contain realistic questions that clinicians and patients would want answered from EHR data. To create realistic questions while constructing the EHRSQL dataset [36], a poll was created at a hospital to gather real-world questions that are frequently asked on the structured EHR data. The Discharge Summary Clinical Questions dataset [43] also included clinically relevant questions by collecting questions that physicians could ask. This ensured the use of medically relevant questions in the EHR QA datasets.

Adding more paraphrases to the QA dataset is another manner to ensure the questions are realistic. This is because, in a real-world scenario, the same question may be posed or stated in different manners. Generation of paraphrases may be machine-generated, human-generated [26], or it could be a combination of both [36]. Table 5 lists the number of paraphrases used per template in different EHR QA datasets.

**Table 5:** Summary of paraphrases used in various electronic health record (EHR) question answering (QA) datasets.

Dataset	Paraphrases per question type, mean	Method of generating paraphrases	Number of questions
MIMICSQL [5]	1	Human labor (crowdsourcing)	10,000
emrQA [26]	7	Human labor (templates generated by physicians were slot-filled)	1,000,000
emrKBQA [8]	7.5	Human labor (templates generated by physicians were slot-filled)	940,173
EHRSQL [36]	21	Human labor and machine learning	24,000

### Open Issues and Future Work

#### Redundancy in the Types of Clinical Questions

Most of the existing EHR QA datasets are template-based datasets that are obtained by slot-filling. These datasets have several instances of the same type of templates that are slot-filled with various entities. Therefore, there is redundancy in the diversity of questions generated. This is still an ongoing issue that needs to be addressed.

#### Need for Multimodal EHR QA Systems

Clinical EHRs contain a vast amount of patient information. Structured EHR data contain highly complementary data that may or may not be present in the clinical notes. The information in structured and unstructured EHR data may contain information that is similar, may contradict, or can provide additional context between these sources. There is a clear need for EHR QA systems that reason across both types of data.

DrugEHRQA [25] and MedAlign [49] datasets are the only multimodal EHR QA datasets (although MedAlign dataset is technically a pseudo-multimodal EHR QA dataset because the QA pairs of the MedAlign dataset are based on an XML markup that are derived from structured and unstructured EHR data). Bardhan et al [25] introduced a simple baseline QA model for multimodal EHR data, and further research is needed to develop a multimodal QA model that unifies the EHR data modalities to obtain a contextualized answer.

#### QA of EHRs on Unseen Paraphrased Questions

QA models trained on clinical question-answer pairs when tested on unseen paraphrased questions have historically produced poor results. There have been works that have tried to address this challenge. The model in Raghavan et al [8] uses paraphrasing detection and generation as a supplementary task to handle this issue. Another solution was discussed in Rawat et al [60]. Rawat et al [60] introduced a multitask learning approach where extractive QA and prediction of answer span were the primary tasks, with an auxiliary task of logical form prediction for the questions. However, this is still an ongoing issue that needs further work.

#### QA of EHRs on Unseen Data

QA models should be able to generalize to new clinical contexts and EHR questions. To study generalization, Yue et al [46] evaluated the performance of a model trained on the emrQA dataset on a new set of questions based on clinical notes of MIMIC-III. The experiment proved that the accuracy of the QA model dropped down by 40% when tested on unseen data. The same research group later proposed a solution [42]. They developed the CliniQG4QA framework, which uses question generation to obtain QA pairs for unseen clinical notes and strengthen QA models without the need for manual annotations. This was done using a sequence-to-sequence-based question phrase prediction model.

This issue was also addressed in question-to-SQL tasks for table-based EHR QA. Tarbell et al [71] introduced the MIMICSQL 2.0 data split (derived from the existing MIMICSQL dataset [5]) to test the generalizability of existing text-to-SQL models on EHRs. The performance of the TREQS [5] model on the MIMICSQL 2.0 data split was drastically poor (logical form accuracy of 0.068 and execution accuracy of 0.173 when trained on paraphrased questions and tested on paraphrased questions), thus showing the need for improvement. To improve generalizability of text-to-SQL tasks on EHR data, Tarbell et al [71] then introduced the use of the T5 model with the data augmentation method using back-translation and further adding out-of-domain training data to improve generalizability on text-to-SQL tasks. The proposed model, even though it outperformed the TREQS model (logical form accuracy of 0.233 and execution accuracy of 0.528 when trained on paraphrased questions and tested on paraphrased questions), still needs further improvement. More work is required in the future to overcome this challenge.

### Progress of QA Models in Real Clinical Applications

Integrating QA systems into clinical workflows allows health care practitioners to access current medical information and recommendations, potentially lowering medical errors and improving patient care. Studies are now being conducted on QA models to determine their accuracy, safety, and reliability in clinical settings. These studies are critical for establishing their usefulness in real-world settings [87]. Efforts are underway to create user-friendly interfaces that allow health care providers to communicate more easily. Some QA models are being tested in cohort selection studies [13] and clinical trials to determine their efficacy and safety in real-world contexts. Deploying QA models in clinical contexts involves ethical problems about patient privacy, bias reduction, and transparency in decision-making. Addressing these concerns is critical for establishing acceptance among health care professionals and patients. To summarize, while QA models have considerable benefits for clinical practice and research, their implementation in real-world clinical applications necessitates resolving integration and ethical issues. To completely harness the power of QA models in health care, artificial intelligence researchers and physicians must keep working together.

### Strengths

In this study, we presented the first scoping review for QA in EHRs. We methodologically collected and screened papers related to EHR QA from January 1, 2005, to September 30, 2023, and performed a thorough review of the existing studies on EHR QA. Then, we explored all the existing datasets, approaches, and evaluation metrics used in EHR QA. Furthermore, we identified the different modalities for QA over EHRs and described the approaches used for each. We have fulfilled all PRISMA-ScR (Preferred Reporting Items for Systematic Reviews and Meta-Analyses extension for Scoping Review) requirements.

This review helps to identify the challenges faced in EHR QA. In addition, this study sheds light on the problems that have been solved along with the additional gaps that are still remaining. This will encourage researchers in this domain to pursue these open problems that have not yet been solved.

### Limitations

Despite the strengths of this study, we note a few limitations. First, the search process was limited to a handful of EHR and QA-related keywords. There is a long tail in how these types of systems are described in the literature, but there is a possibility that we might have missed relevant studies that did not match this initial search criteria. We used forward snowballing to partially resolve this issue. This helped us to identify 10 additional papers that we had missed out on earlier. However, despite this, there is still a slim chance that we might have missed a few relevant studies in our final list. Furthermore, given the current expansion of research into EHR QA, we predict that new studies will be added to this list since our search.

### Conclusions

In recent years, QA over EHRs has made significant progress. This is the first systematic or scoping review of QA over EHRs. In this paper, we have provided a detailed review of the different approaches and techniques used for EHR QA. The study began by discussing the need for large domain-specific EHR QA datasets and then discussed the existing EHR QA datasets. We have reviewed the different unimodal EHR QA models used for both structured EHRs and unstructured EHRs, as well as QA models on multimodal EHRs. Then, we identified the major challenges in this field, such as the limited number of clinical annotations available for EHR QA dataset generation. We also talked about potential future directions in this field. It is a relatively new field with many unexplored challenges that require attention. This study should help future researchers explore various research directions within EHR QA and expand the horizons of research areas in this field.

## Appendix

Multimedia Appendix 1 PRISMA (Preferred Reporting Items for Systematic Reviews and Meta-Analyses) checklist.

Multimedia Appendix 2 Summaries of selected papers.

Multimedia Appendix 3 Comparison of different electronic health record question answering datasets. The column “Database/Corpus” refers to the electronic health record database or clinical annotations on which the question answering datasets are based.

Multimedia Appendix 4 Summary of electronic health record question answering (QA) models. The model’s task can be of type machine-reading comprehension (MRC), KBQA, “question to SQL query,” and “question to SPARQL.” The “Answer type” column specifies the expected type of the electronic health record-QA model, indicating whether the answer is derived from tables, text notes, a knowledge graph, or a combination of sources. “Dataset”’ refers to the dataset used to evaluate the QA model.

Multimedia Appendix 5 Evaluation metrics used for evaluating different electronic health record-question answering models.

## Abbreviations

BERT: Bidirectional Encoder Representations from Transformers
BLEU: Bilingual Evaluation Understudy
ClinicalKB: Clinical Knowledge Base
EHR: electronic health record
KB: knowledge base
LSTM: long short-term memory
MedTS: Medical text-to-SQL
METEOR: Metric for Evaluation of Translation with Explicit Ordering
MIMIC: Medical Information Mart for Intensive Care
MRC: machine-reading comprehension
n2c2: natural language processing clinical challenge
NLP: natural language processing
PRISMA: Preferred Reporting Items for Systematic Reviews and Meta-Analyses
PRISMA-ScR: Preferred Reporting Item for Systematic Reviews and Meta-Analyses extension for Scoping Review
QA: question answering
SPARQL: SPARQL Protocol and RDF Query Language
SQuAD: Stanford QA dataset
TER: translation error rate
TREQS: Translate-Edit Model for Question-to-SQL
UMLS: Unified Medical Language System
