# Supplementary material for: Question Answering for Electronic Health Records: Scoping Review of Datasets and Models
Source: J Med Internet Res. 2024 Oct 30;26:e53636. doi: 10.2196/53636 (PMC11561445; doi:10.2196/53636)
Supplement: Multimedia Appendix 3 [file jmir_v26i1e53636_app3.docx]

Multimedia Appendix. Comparison of different EHR QA datasets. The column "Database/Corpus" refers to the EHR database or clinical annotations on which the QA datasets are based.

| Dataset | Number of Questions | EHR Modality | Database/ Corpus | Example |
| --- | --- | --- | --- | --- |
| emrQA [26] | 455,837 QA pairs and 1,295,814 question-logical form pairs | Clinical notes (mostly discharge summaries) | n2c2 annotations | Question: Has the patient ever had an abnormal BMI  Answer: BMI: 33.4 Obese, high risk |
| RxWhyQA [27] | 96,939 QA pairs | Clinical notes (discharge summaries) | National NLP Clinical Challenges (n2c2) corpus of 2018 | Question: Why was the patient prescribed Metoprolol?  Answer 1: Atrial fibrillation  Answer 2: Hypertension |
| Raghavan et al. (2018) [34] | 1747 QA pairs | Clinical notes | Cleveland Clinic (medical records) | Question: Why does the patient take trazodone?  Answer: Insomnia |
| Fan et al. (2019) [35] | 277 sentences containing explicit why-QA cues | Clinical notes (discharge summaries) | 2010 n2c2/VA NLP challenge | Question: Why was the Bactrim for urinary tract infection stopped?  Answer: diarrhea |
| RadQA [37] | 6148 question-answer evidence triplets | Clinical notes (radiology reports) | MIMIC-III | Question: What is the position of central line?  Answer: Left internal jugular line at the brachiocephalic vein junction |
| Oliveira et al. (2021) [38] | 18 questions  (10 questions based on nursing notes and eight questions based on medical notes) | Nursing and medical notes | SemClinBr corpus  (Portuguese nursing  and medical notes) | Question: Foram solicitados exames?  (In Portuguese) |
| Yue et al. (2021) [42,74] | 1287 QA pairs | Clinical notes | MIMIC-III | Question: Why did the patient get abd pain?  Answer: 5. abd pain: suspect secondary to chronic pancreatitis. |
| Discharge Summary Clinical Questions (DiSCQ) [43] | 2029 questions | Clinical notes (discharge summaries) | MIMIC-III | Question: Date of diagnosis? Any interventions done (RT, surgery)?  Trigger: prostate cancer, benign prostatic hypertrophy |
| Mishra et al. (2021) [45] | 6 questions/ article | Clinical notes (discharge summaries) | MIMIC-III | Question: Does the patient have CVA?  Related context:  Context Initial Segment - Discharge Diagnosis  Context end segment – Discharge Condition |
| Yue et al. (2020) [46] | 50 QA pairs | Clinical notes | MIMIC-III | Question 1: How was the diagnosis of acute cholecystitis made?  Question 2: Was an edema found in the physical exam? |
| CLIFT [47] | 7.5 K question -answer pairs | Clinical notes | MIMIC-III | (Not available) |
| Hamidi and Roberts (2023) [48] | 15 question-answer pairs | Clinical notes | MIMIC-III | (Not available) |
| Mahbub et al. (2023) [50] | 28855 question-answer pairs | Clinical progress notes | VA Corporate Data Warehouse (CDW) | Question: Which IV drugs has the pt used?  Answer: hx of iv heroin use most recently >2 days ago |
| Dada et al. (2023) [51] | 29,273 question-answer pairs | Radiology reports | Radiology reports related to brain CT scans | (Not available) |
| MIMICSQL [5] | 10,000 question-SQL query pairs | Structured tables | MIMIC-III | Question: How many female patients underwent the procedure of abdomen artery incision?  SQL query: select count (distinct demographic."subject_id") from demographic inner join procedures on demographic.hadm_id = procedures.hadm_id where demographic."gender" = "f" and procedures."short_title" = "abdomen artery incision" |
| emrKBQA [8] | 940,000 question - logical form - answer triplets | Structured tables | MIMIC-III | Question Template: “Is the patient on \|medication\|?”  Logical Form: “MedicationEvent \|medication\|”  Question Form Template: What were the results of abnormal \|test\| in \|date\|?  Logical form: LabEvent(\|test\|) [abnormalResultFlag=Y, date=\|date\|, result=x] OR [LabEvent(\|test\|) [date=\|date\|, abnormalResultFlag=Y] |
| Roberts et al. (2016) [23] | 446 question – logical form pairs | Structured tables | - | Question: What is his emotional status? |
| Roberts et al. (2015) [24] | 100 question – logical form pairs | Structured tables | - | Question: What are the positive tests?  Logical form: positive(λx.has test(x, C0022885, visit)) |
| EHRSQL [36] | 24,000 question-SQL query pairs | Structured tables | MIMIC-III and eICU | NL Question: tell me the length of the hospital stay of the last hospital stay of patient 3745.  SQL query: select strftime(’%J’,admissions.dischtime) - strftime(’%J’,admissions.admittime) from admissions where admissions.subject_id = 3745 and admissions.dischtime is not null order by admissions.admittime desc limit 1 |
| Soni et al. (2019) [44] | 1000 question -logical form-answer triplets | Structured tables | Synthea generated FHIR server | Question: What was the highest hemoglobin A1c value in the past 2 years?  Logical Form: max(λx.has_concept(x, C0366781) ^ time_within(x, ‘past 2 years’)) |
| Kim et al. (2022) [39] | 168,574 question-program pairs | Knowledge graph | MIMIC-III | Question: “provide the procedure short title and drug name of patient id 23.”  Program:  <r1>=gen_entset_down('/subject_id/23','/hadm_id')<exe>  <r2>=gen_entset_down(<r1>,'/procedures')<exe>  <r3>=gen_entset_down(<r2>,'/procedures_icd9_code')<exe>  <r4>=gen_litset(<r3>,'/procedures_short_title')<exe>  <r5>=gen_entset_down(<r1>,'/prescriptions')<exe>  <r6>=gen_litset(<r5>,'/drug')<exe>  <r7>=concat_litsets(<r4>,<r6>)<exe>  Answer: : [“percutan aspiration gb”, “ciprofloxacin iv”] |
| ClinicalKBQA [40] | 9,000 QA pairs | Knowledge graph | Annotations of n2c2 dataset used to generate knowledge graph. | Question 1: What medications has patient P939003 ever been prescribed?  Question2: When was patient P32 discharged?  Question3: Why is patient P280639 on coumadin? |
| MIMICSPARQL* [41] | 10,000 question -query pairs | Knowledge graph | MIMIC-III | Question: Calculate the maximum age of patients who have arrhythmia primary disease and were born after the year 2080?  SQL query: SELECT (MAX (?age) as ?agg ) WHERE{?subj_id?adm_id. ?adm_id ?age. ?adm_id “Arrhythmia”. ?subj_id ?dob. filter ( ?dob > 2080-01-01 )} |
| DrugEHRQA [25] | 70,000 QA pairs | Structured tables and clinical notes | MIMIC-III | Question : What is the dosage of Guaifenes in prescribed to the patient with admission id 174037  Multi-modal answer: 5-10 mls |
| MedAlign [49] | 983 question-instruction pairs and 303 question-instruction-answer triplets | Response based on XML markup derived from structured and unstructured EHR data | Hospital database | Instruction: Summarize from the EHR the strokes that the patient had and their associated neurologic deficits.  Answer: The patient had strokes in the L basal ganglia in 2018 and multiple strokes in 2022: R occipital, left temporal, L frontal. The patient had right sided weakness associated with the 2018 stroke after which she was admitted to rehab. She then had a left sided hemianopsia related to the 2022 stroke. |
