# Supplementary material for: Question Answering for Electronic Health Records: Scoping Review of Datasets and Models
Source: J Med Internet Res. 2024 Oct 30;26:e53636. doi: 10.2196/53636 (PMC11561445; doi:10.2196/53636)
Supplement: Multimedia Appendix 4 [file jmir_v26i1e53636_app4.docx]

Multimedia Appendix. Summary of EHR QA models. The model’s task can be of type machine-reading comprehension (MRC), KBQA, “question to SQL query,” and “question to SPARQL.” The ‘Answer Type’ column specifies the expected type of the EHR-QA model, indicating whether the answer is derived from tables, text notes, a knowledge graph, or a combination of sources. ‘Dataset’ refers to the dataset used to evaluate the QA model.

| Papers | Task | Answer Type | Dataset |
| --- | --- | --- | --- |
| Pampari et al. (2018) [26] | MRC | Text span | emrQA |
| Moon et al. (2023) [27] | MRC | Text span | RxWhyQA |
| Oliveira et al. (2021) [38] | MRC | Text span | SQUAD dataset in Portuguese and another QA dataset developed in Portuguese from SemClinBr corpus |
| Yue et al. (2021) [42] | MRC | Text span | emrQA and [42, 74] as test set. |
| Hamidi et al. (2023) [48] | MRC | Text span | QA dataset constructed based on TREC 2016 Clinical Decision Support Track |
| Fleming et al. (2023) [49] | Multi-step refinement approach using standard prompt template | Response based on XML markup derived from EHR data | MedAlign dataset |
| Mahbub et et al. (2023) [50] | MRC | Text span | QA dataset on Injection Drug use constructed |
| Dada et al. (2023) [51] | MRC | Text span | Reading comprehension question answering constructed based on radiology report |
| Roberts et al. (2017) [57] | question to logical form | Text span | Annotation of 446 questions in [[23](#Rob98)] |
| Rawat et al. (2019) [59] | MRC | Text span | Naranjo Scale Questionnaire |
| Rawat et al. (2020) [60] | MRC | Text span | emrQA and MADE-QA dataset |
| Wen et al. (2020) [64] | MRC | Text span | n2c2 notes, emrQA_why_, and SQuAD_why_ |
| Soni et al. (2020) [65] | MRC | Text span | CliCR and emrQA dataset |
| Mairittha et al. (2020) [66] | MRC | Text span | why-question answering (why-QA) dataset developed based on 2010 n2c2/VA Workshop on Natural Language Processing Challenges for Clinical Records |
| Moon et al. (2022) [67] | MRC | Text span | Why-QAs from the n2c2 ADE Challenge and Medication Why-QAs from the emrQA |
| Li et al. (2023) [68] | MRC | Text span | emrQA |
| Yang et al. (2022) [69] | MRC | Text span | emrQA |
| Lehman et al. (2023) [73] | MRC | Text span | RadQA |
| Kang and Baek et al. (2022) [70] | Knowledge conditioned Feature Modulation on Transformer for MRC | Text span | emrQA |
| Wang et al. (2020) [5] | question to SQL query | Table content | MIMICSQL |
| Raghavan et al. (2021) [8] | question to logical forms | Table content | emrKBQA |
| Pan et al. (2021) [62] | question to SQL query | Table content | MIMICSQL |
| Soni et al. (2022) [63] | question to logical forms | Table content | ICU_data_ [[23](#Rob98)] and FHIR_data_ [[44](#Son18)] |
| Tarbell et al. (2023) [71] | question to SQL query | Table content | MIMICSQL 2.0 split |
| quEHRy [72] | question to answer extraction pipeline | Table content | FHIR_data_ [[44](#Son18)] and ICU_data_ [[23](#Rob98)] |
| Kim et al. (2022) [39] | question to Program | Element from knowledge graph | MIMICSPARQL* |
| Wang et al. (2021) [40] | KBQA | Element from knowledge graph | ClinicalKBQA |
| Park et al. (2021) [41] | question to SPARQL query | Element from knowledge graph | MIMICSPARQL* |
| Schwertner et al. (2019) [58] | question to SPARQL query | Element from knowledge graph | QA dataset developed on Oncology XML EHR notes in Portuguese |
| Bae et al. (2021) [61] | question to query (SQL/ SPARQL) | Table content or element from knowledge graph | MIMICSQL* and MIMICSPARQL* |
| Bardhan et al. (2022) [25] | Multimodal QA | Text span or Table content | DrugEHRQA |
