# Supplementary material for: Question Answering for Electronic Health Records: Scoping Review of Datasets and Models
Source: J Med Internet Res. 2024 Oct 30;26:e53636. doi: 10.2196/53636 (PMC11561445; doi:10.2196/53636)
Supplement: Multimedia Appendix 5 [file jmir_v26i1e53636_app5.docx]

Multimedia Appendix. Evaluation metrics used for evaluating different EHR-QA models.

| Papers | Task | Answer Type | Evaluation Metric |
| --- | --- | --- | --- |
| Pampari et al. (2018) | MRC | Text span | Exact Match and F1 score |
| Moon et al. (2023) | MRC | Text span | F1 score |
| Oliveira et al. (2021) | MRC | Text span | Precision, Recall, F1 score |
| Yue et al. (2021) | MRC | Text span | Exact Match and F1 score |
| Yue et al. (2020) | MRC | Text span | Exact Match and F1 score |
| Hamidi et al. (2023) | MRC | Text span | Accuracy, Relevance, Coverage, and Coherence |
| Mahbub et et al. (2023) | MRC | Text span | Exact Match, Perfect Recall, and F1 Score |
| Dada et al. (2023) | MRC | Text span | Exact match and F1 score |
| Roberts et al. (2017) | NL question to logical form | Text span | Accuracy |
| Rawat et al. (2019) | MRC | Text span | Weighted, macro-averaged and micro-averaged Precision, Recall and F-score |
| Rawat et al. (2020) | MRC | Text span | Exact Match and F1 score |
| Wen et al. (2020) | MRC | Text span | Exact Match and partial match |
| Soni et al. (2020) | MRC | Text span | Exact Match and F1 score |
| Mairittha et al. (2020) | MRC | Text span | Exact Match and F1 score |
| Moon et al. (2022) | MRC | Text span | Exact Match and Partial Match |
| Li et al. (2023) | MRC | Text span | Exact Match and F1 score |
| Yang et al. (2022) | MRC | Text span | Exact Match and F1 score |
| Lehman et al. (2023) | MRC | Text span | Exact match and F1 score |
| Kang and Baek et al. (2022) | Knowledge conditioned Feature Modulation on Transformer for MRC | Text span | Exact Match and the F1 score |
| Wang et al. (2020) | NL Question to SQL query | Table content | Logical form accuracy and execution accuracy |
| Raghavan et al. (2021) | NL Question to logical form | Table content | Exact Match and Denotation Accuracy |
| Pan et al. (2021) | NL question to SQL query | Table content | Logical form accuracy and execution accuracy |
| Tarbell et al. (2023) | NL Question to SQL query | Table content | Logical form accuracy and execution accuracy |
| Kim et al. (2022) | NL Question to Program | Element from knowledge graph | Execution accuracy |
| Wang et al. (2021) | KBQA | Element from knowledge graph | Precision, recall, accuracy, micro-F1, and macro-F1 |
| Park et al. (2021) | NL question to SPARQL query | Element from knowledge graph | Logical form accuracy, execution accuracy, and structural accuracy |
| Bae et al. (2021) | NL Question to Query (SQL/SPARQL) | Table content or element from knowledge graph | Logical form accuracy, execution accuracy, and structural accuracy |
